# Detection of SARS-CoV-2 in a dog with hemorrhagic diarrhea

**DOI:** 10.1186/s12917-022-03453-8

**Published:** 2022-10-12

**Authors:** Miguel Padilla-Blanco, Santiago Vega, Luis Enjuanes, Alfonso Morey, Teresa Lorenzo, Clara Marín, Carmen Ivorra, Elisa Maiques, Vicente Rubio, Consuelo Rubio-Guerri

**Affiliations:** 1 Departamento de Farmacia, Facultad de Ciencias de la Salud, Universidad Cardenal Herrera-CEU, 46113 Alfara del Patriarca, Valencia Spain; 2 Departamento de Producción y Sanidad Animal, Salud Pública Veterinaria y Ciencia y Tecnología de los Alimentos, Instituto de Ciencias Biomédicas, Facultad de Veterinaria, Universidad Cardenal Herrera-CEU, 46115 Alfara del Patriarca, Valencia Spain; 3 Centro Nacional de Biotecnología (CNB-CSIC), Campus de Cantoblanco, Universidad Autónoma de Madrid, 28049 Madrid, Spain; 4 Clínica Veterinaria Morey, 51004 Ceuta, Spain; 5 Departamento de Ciencias Biomédicas, Facultad de Ciencias de la Salud, Universidad Cardenal Herrera-CEU, Alfara del Patriarca, 46113 Madrid, Spain; 6 I+D+I Department, Sequencing Multiplex SL (I+D+I, Seqplexing), Parque Científico Universidad de Valencia, 46980 Paterna, Valencia Spain; 7 Instituto de Biomedicina de Valencia del Consejo Superior de Investigaciones Científicas (IBV-CSIC) and CIBERER-ISCIII, 46010 Valencia, Valencia Spain

**Keywords:** SARS-CoV-2, B.1.177, Ile402Val S protein substitution, Dog COVID-19, Zoonosis, One Health

## Abstract

**Background:**

Severe acute respiratory syndrome coronavirus 2 (SARS-CoV-2), the causative agent of COVID-19, has infected several animal species, including dogs, presumably via human-to-animal transmission. Most infected dogs reported were asymptomatic, with low viral loads. However, in this case we detected SARS-CoV-2 in a dog from the North African coastal Spanish city of Ceuta presenting hemorrhagic diarrhea, a disease also reported earlier on in an infected dog from the USA.

**Case presentation:**

In early January 2021, a West Highland Terrier pet dog from Ceuta (Spain) presented hemorrhagic diarrhea with negative tests for candidate microbial pathogens. Since the animal was in a household whose members suffered SARS-CoV-2 in December 2020, dog feces were analyzed for SARS-CoV-2, proving positive in a two-tube RT-PCR test, with confirmation by sequencing a 399-nucleotide region of the spike (*S*) gene. Furthermore, next-generation sequencing (NGS) covered > 90% SARS-CoV-2 genome sequence, allowing to classify it as variant B.1.177. Remarkably, the sequence revealed the Ile402Val substitution in the spike protein (S), of potential concern because it mapped in the receptor binding domain (RBD) that mediates virus interaction with the cell. NGS reads mapping to bacterial genomes showed that the dog fecal microbiome fitted best the characteristic microbiome of dog’s acute hemorrhagic diarrhea.

**Conclusion:**

Our findings exemplify dog infection stemming from the human SARS-CoV-2 pandemic, providing nearly complete-genome sequencing of the virus, which is recognized as belonging to the B.1.177 variant, adding knowledge on variant circulation in a geographic region and period for which there was little viral variant characterization. A single amino acid substitution found in the S protein that could have been of concern is excluded to belong to this category given its rarity and intrinsic nature. The dog’s pathology suggests that SARS-CoV-2 could affect the gastrointestinal tract of the dog.

**Supplementary Information:**

The online version contains supplementary material available at 10.1186/s12917-022-03453-8.

## Background

Severe acute respiratory syndrome coronavirus 2 (SARS-CoV-2) emerged on a wholesale market in Wuhan (China) on December, 2019 [1]. It is assumed to be a zoonotic pathogen that could have originated in non-clarified wild animals and transmitted to humans via a yet undetermined intermediate host [2], which led to a global pandemic through human-to-human transmission [3]. In addition to humans, human-related infections have been reported in several carnivore species of domestic pets and captive animals, including several species of felines (cats, tigers, lions, snow leopards, a cougar, an Amur leopard and a fishing cat) and mustelids (ferrets, American mink and Asian small-clawed otters), dogs, spotted hyenas and a binturong [4]. SARS-CoV-2 was also detected in omnivorous and herbivorous zoo animals, such as gorillas, a South American coati, hippos [4] and Antillean manatees [5]. It is interesting to note that it has also reached non-captive animals, having been detected in free-ranging white-tailed deer [6], feral American mink [7] and European wild otter [8].

Among pets, ferrets and cats appear to be those most vulnerable to contagion [9]. Natural infections of these animals in most cases were asymptomatic or caused mild disease [10–13]. However, a cat was reported [14] to develop more serious disease, with severe respiratory distress and thrombocytopenia, and had to be humanely euthanized. Regarding dogs, their susceptibility to SARS-CoV-2 infection is considered lower than that of felines due to poor viral replication in this species [9]. The first two dogs reported [15] as SARS-CoV-2-positive were detected early in the COVID-19 pandemic, in Hong Kong, living with infected people. They were asymptomatic, had relatively low viral loads, and their viral sequences were identical to those found in their respective owners. Later on, other dogs infected with prevalent SARS-CoV-2 variants have been detected throughout the world [4]. As already indicated, the dogs generally were asymptomatic or presented mild disease, although respiratory, gastrointestinal and cardiac complication, sometimes severe, have also been reported in these carnivores [16–21]. Of interest because of the symptomatic similarity with the present case, was the finding of hemorrhagic diarrhea in an infected dog in the USA with low viral load (13 copies RNA/μl) [16].

We now report a dog with hemorrhagic diarrhea presenting SARS-CoV-2 infection revealed by RT-PCR, Sanger DNA sequencing of a partial 399-nt region of the *S* gene for the SARS-CoV-2 spike protein, and next generation sequencing (NGS) of >90% of the viral genome, which allowed its identification as belonging to the B.1.177 variant. Interestingly, both sequencing methodologies revealed the Ile402Val substitution in the S protein sequence, of potential concern since it affected the Receptor Binding Domain (RBD) of S. In addition, NGS reads for genomes of fecal bacteria have also provided insight on the fecal microbiome of this dog, which agrees with that for canine acute hemorrhagic diarrhea [22].

## Case presentation

In early January 2021, a well-cared properly vaccinated and regularly wormed 11-year-old West Highland Terrier pet dog in a family in the North African Autonomous Spanish city of Ceuta started producing pasty stools suggestive of mucous enteritis which, after a few days, transformed into persistent mucohemorrhagic enteritis with pasty bloody stools. Physical examination was unremarkable and did not substantiate altered body temperature, the presence of swollen lymph nodes or pain upon abdominal palpation, while cardiac and pulmonary auscultation were normal. Complete blood cell count (CBC) and routine blood biochemistry were uninformative, and stool examination and analysis did not reveal gross steatorrhea, amylorrhea, or the presence of common parasites (*Giardia*, nematodes, cestodes or coccidia). Colonoscopy showed a moist pink mucosa, with a capillary refill time (CRT) < 2 seconds and no ulcers, tumors or other gross macroscopic pathological alterations. Histopathological analysis of a mucosal biopsy sample revealed areas of superficial erosion of the epithelium lining the lumen and a moderate inflammatory infiltrate throughout the lamina propria and between the intestinal crypts of the colic mucosa. The infiltrating cells were essentially mononuclear, largely lymphocytes with some plasma cells, leading to the histopathological diagnosis of lymphoplasmacytic colitis of grade II with superficial focal erosions.

**Fig. 1:**
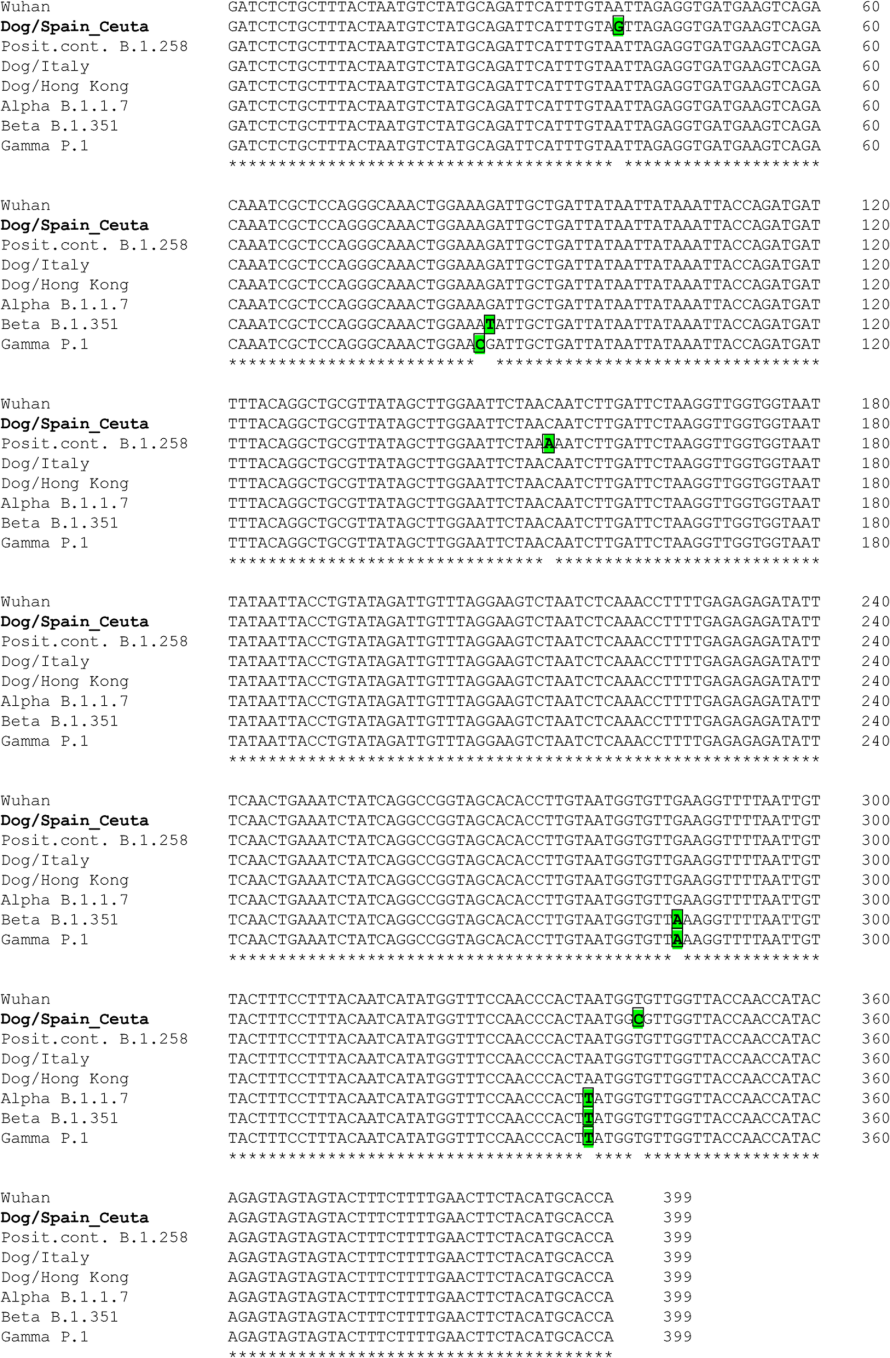
Alignment of the partial *S* gene sequences. The sequences shown are, from top to bottom, the following ones: consensus early Wuhan (GenBank ID: NC_045512.2); present dog fecal sequence (in bold; GenBank and GISAID IDs: MW872017 and EPI_ISL_1490671, respectively); the positive control used (GISAID ID: EPI_ISL_433320); the variants indicated with their trivial and canonic designations (Alpha, GISAID ID: EPI_ISL_581117; Beta, GISAID ID: EPI_ISL_660605; Gamma, GISAID ID: EPI_ISL_792680). Bases exhibiting complete conservation are marked at the bottom by asterisks. The base deviations from the Wuhan sequence are green-shadowed and squared. The alignment was performed using Bio-Edit [23]

**Fig. 2:**
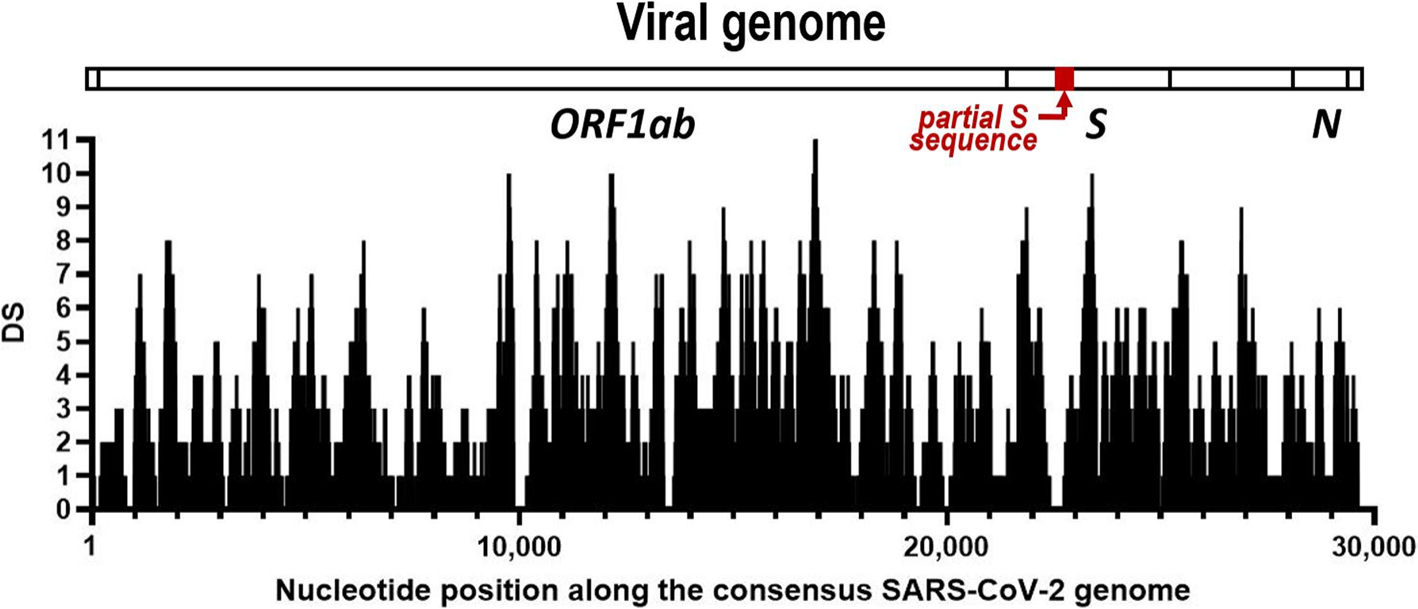
SARS-CoV-2 genomic coverage obtained by Next-generation sequencing (NGS). The horizontal axis corresponds to the position of the SARS-CoV-2 consensus genome and the vertical one to the depth of the sequence (DS) at a given position. On the top, aligned over the nucleotide positions, the bar shows a linear diagram of the entire genome, showing the span of the *ORF1ab*, *spike* (*S)* and *nucleocapsid (N*) genes as well as the region amplified of the *S* gene that was subjected to Sanger sequencing (in black, labeled “partial S sequence”)

Since the dog was in close contact with human household members who suffered symptomatic and RT-PCR-proven COVID-19 at the end of December 2020, a stool sample from the dog was used for SARS-CoV-2 assay. Total fecal RNA was isolated (see Supplementary Methods) and was used first in a one-step commercial SARS-CoV-2 RT-PCR test (Viasure, CerTest Biotec, Zaragoza, Spain) with negative results. It was also used in a more sensitive two-tube RT-PCR assay ([7] and Supplementary Methods) whose results were consistently and repeatedly positive. RNA from the stools from other four dogs studied in parallel were negative in both assays. These results replicated our own previous findings [7] with two feral mink in which the commercial test was negative but the two-tube test was positive. This two-tube assay focuses on a partial 399 base pairs (bp) sequence of the SARS-CoV-2 viral spike glycoprotein gene (*S*) (Figs. 1 and 2, top). The low load of viral RNA reflected in the high Ct value (average of 32.95 in six different repeats of the test, three of them on each one of two separate RNA extractions and retrotranscription assays from the same stool) was consistent with the negativity of the commercial test, as previously experience in mink [7]. The positivity of the two-tube test was corroborated physically by subsequent visualization by agarose gel electrophoresis of the amplified product, which was of the expected size, and by Sanger sequencing, which yielded the expected sequence for the intended amplicon (Figs. 1 and 2, top).

Alignment with the SARS-CoV-2 consensus sequence (GenBank ID NC_045512.2) of the partial *S* gene sequence obtained from our dog (GenBank accession number MW872017) revealed two nucleotide changes relative to the reference sequence (Wuhan original sequence) (Fig. 1). These changes, at positions 40 and 342 of the amplicon, were the A>G and T>C transitions (genome nucleotides 22766 and 23068, Figs. 1 and 2, top; *S* gene coding sequence positions 1204 and 1506, at codons 402 and 502, respectively). Only the first of these two substitutions was non-synonymous, causing the amino acid change Ile402Val in the S protein, a change that maps in the receptor binding domain (RBD) of this protein. The RBD is the part of the spike protein that interacts with the angiotensin-converting enzyme 2 (ACE2) cell receptor for SARS-CoV-2 [24], stressing the possibility that amino acid changes in this domain could affect the ability of the virus to infect cells.

Despite de low Ct value most likely reflecting a low viral load in the analyzed fecal sample, we attempted NGS on the isolated RNA (see Supplementary Methods). This approach yielded 623,796 reads, but, after application of the quality and sequence length filters indicated in the Supplementary Methods, and following removal of 54,085 reads that mapped with the dog genome, 116,637 high-quality reads remained (given the high number of small reads). These high-quality reads reflected the entire population of microorganisms present in the stool sample, with only 1,290 of them mapping to the SARS-CoV-2 consensus genome (Fig. 2). Independently of the sequencing depth (number of times each position was found in the SARS-CoV-2 mapped reads) these reads covered 90.6% of the entire viral genome, with just 2,813 positions of the 29,903 nucleotides of the whole genome not covered. Bioinformatic analysis revealed a number of nucleotide substitutions relative to the consensus SARS-CoV-2 genome sequence (Table 1). Submission of the sequence to GISAID (https://www.gisaid.org/) resulted in the assignment of the virus to the B.1.177 variant. Indeed, the sequence hosted the three characteristic mutations of this variant [25], Ala222Val, Ala220Val and Val30Leu in the S, N and 10 proteins, respectively (Table 1). It is to be stressed that the two nucleotide substitutions detected by Sanger sequencing (see above) were also detected by NGS.

**Table 1:** Base substitutions and corresponding amino acid substitutions in the genome of the SARS-CoV-2 virus found in the infected dog of this study

Genome Position^a^	Gene	Base change^b^	Protein	Non-Synonymous substitution
241	*5' UTR*	C > T	-	-
445	*ORF1ab*	T > C	NSP1	-
3037		C > T	NSP3	-
6286		C > T		-
14408		C > T	NSP12	Pro323Leu
15313		G > A		Ala625Thr
15418		G > T		Ala660Ser
21255		G > C	NSP16	-
21776	*S*	G > C	S	Gly72Arg
22227		C > T		Ala222Val
22766		A > G		Ile402Val
23068		T > C		-
23403		A > G		Asp614Gly
24034		C > T		-
26530	*M*	A > G	M	Asp3Gly
27944	*ORF8*	C > T	8	-
28083		G > T		Glu64STOP
28378	*N*	G > T	N	-
28703		G > C		Asp144His
28932		C > T		Ala220Val
29645	*ORF10*	G > T	10	Val30Leu

^a^Position in the consensus genome (GenBank NC_045512.2)

^b^Substitution relative to the base in the consensus genome. As the sequencing was based on the genome retrotranscribed to DNA, thymine should correspond to uracil in the viral RNA genome

**Fig. 3:**
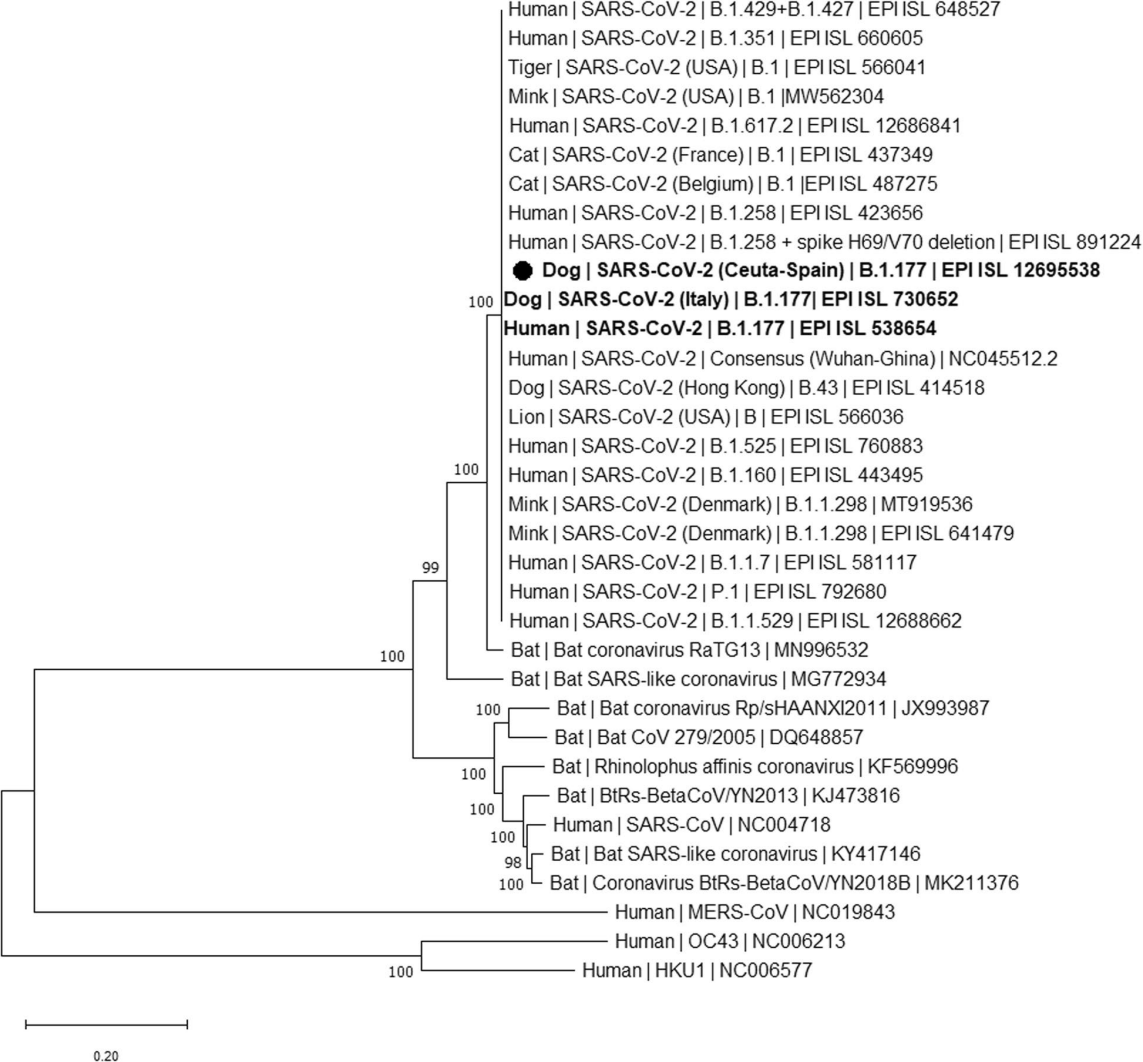
Phylogenetic analysis. Maximum Likelihood Tree based on the complete SARS-CoV-2 genome. The analysis was carried out as reported [7] and is detailed in Supplementary Methods. The evolutionary history was inferred by the Neighbor-Joining method. The optimal tree is shown. The percentage of trees in which the associated taxa clustered together is shown next to branches. The tree is drawn to scale, with branch lengths proportional to the number of substitutions per site. This analysis involved 34 nucleotide sequences. There were a total of 32,122 positions in the final dataset. Evolutionary analyses were conducted in MEGA11 [26]. The name of each sequence is composed of four elements separated by vertical lines: the host where the virus was isolated; the virus strain name (for the SARS-CoV-2 sequences isolated from animals other than humans, countries where they were found are shown in brackets); the specific SARS-CoV-2 variant that they belonged to; and the GenBank or GISAID Accession ID. Bold names indicate sequences belonging to the B.1.177 variant. The black dot (●) marks the present dog SARS-CoV-2 sequence

For completeness of the analysis, we assessed the phylogenetic relation of the near-complete viral genome sequence with those of other SARS-CoV-2 variants that have infected humans, dogs or other animals (Fig. 3). The phylogenetic tree shows that all SARS-CoV-2 variants were included in the same cluster, different from the clusters in which non-SARS-CoV-2 coronavirus sequences were found. Furthermore, our sequence was more closely related to the B.1.177 variant than to other variants, again supporting its classification as belonging to the B.1.177 variant.

On the other hand, the Ile402Val substitution of the S protein, which is not specific of the B.1.177 variant, has been reported in only 316 SARS-CoV-2 sequences of the GISAID database (accessed on May 17, 2022). Fifteen of these occurrences correspond to genomes of the B.1.177.4 variant, which is closely related to the B.1.177 variant, sharing the three specific mutations of this last variant (see above). These B.1.177.4 sequences were collected in England between October and November 2020, before the detection of the virus in our dog (January 2021), but none of them arose from Spain or Morocco (the country of our dog and the land region surrounding Ceuta, respectively).

**Fig. 4:**
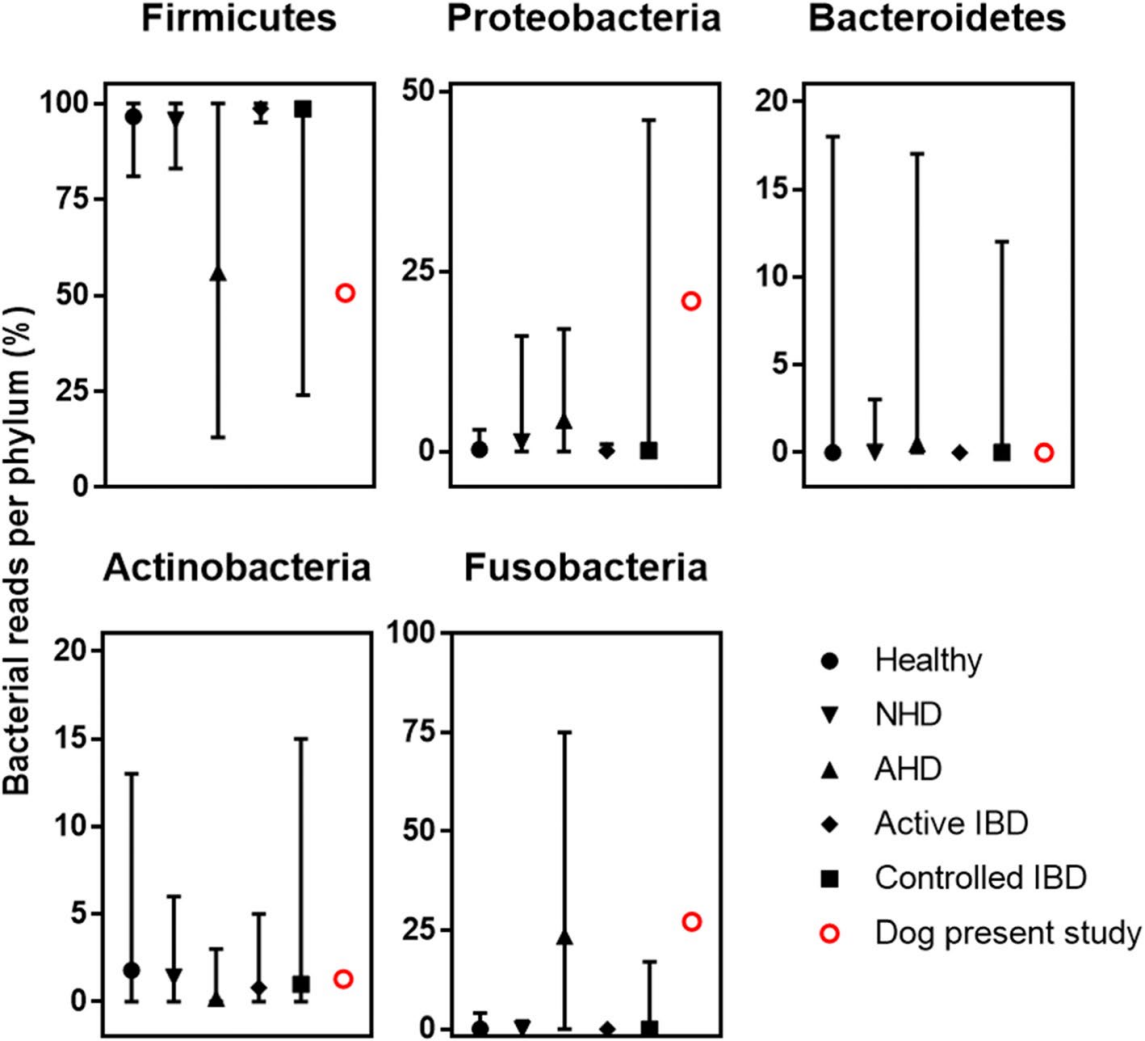
Comparison of fecal abundance of main bacterial phyla in the present dog with the abundances reported [22] in dogs with acute diarrhea and inflammatory bowel disease. For details on microbiome monitoring, see the text and the Supplementary Methods. Data from our dog stool are shown in red. All other results are from [22]. NHD, acute non-hemorrhagic diarrhea (*n* = 12); AHD, acute hemorrhagic diarrhea (*n* = 13); IBD, inflammatory bowel disease (active disease, *n* = 9; therapeutically controlled and thus clinically insignificant, *n* = 10). The symbols give the median for the percent abundance in each dog, and the whiskers give the range

We also exploited the NGS data to estimate the abundance in the stool of bacteria belonging to the five major phyla of Firmicutes, Proteobacteria, Bacteroidetes, Actinobacteria and Fusobacteria. This information could be gathered because the vast majority of the >115,347 high-quality reads remaining after subtraction of the reads mapping to the dog and SARS-CoV-2 genomes were sequences mapping to microbial genomes, mostly to bacterial genomes (see Supplementary Methods). We compared our results (Fig. 4) with those reported by Suchodolski et al. [22] on 32 healthy dogs or on dogs with signs of either acute non-hemorrhagic diarrhea (NHD; *n* = 12), acute hemorrhagic diarrhea (AHD, *n* = 13), active inflammatory bowel disease (Active IBD; *n* = 9), or therapeutically controlled clinically insignificant inflammatory bowel disease (Controlled IBD; *n* = 10). In our dog, the distribution of reads among bacteria pertaining to these phyla, as percentage of recognized reads for bacteria from these phyla, was 50.7, 20.9, 0, 1.3 and 27.1 for Firmicutes, Proteobacteria, Bacteroidetes, Actinobacteria and Fusobacteria, respectively. Except for Proteobacteria, the proportion of reads in our dog closely resembled the median frequency of reads for these phyla in the AHD dog group, clearly distinguishing the intestinal microbiota of our dog from those of healthy dogs, of dogs with non-hemorrhagic diarrhea or of those with active inflammatory bowel disease.

## Discussion and Conclusions

This report supports the notion that dogs can be SARS-CoV-2-infected when intimately related with COVID-19 affected humans [15, 16]. The presentation of clinically relevant hemorrhagic diarrhea, and the previous description of the same pathology in an unrelated dog that was reported to be infected with SARS-CoV-2 [16] raises the possibility that this intestinal alteration, considered related to digestive tract microbial dysbiosis [27], was favored or even caused by SARS-CoV-2. The fact that the proportions of five major bacterial phyla in the fecal microbiome of our dog do not substantially differ from those in dogs with acute hemorrhagic diarrhea [22] does not support the occurrence of a specific fecal microbiome disturbance for SARS-CoV-2 infection. In any case, our results, together with those from another dog with SARS-CoV-2 infection and hemorrhagic diarrhea [16], call for a study of the potential connection between SARS-CoV-2, dog hemorrhagic diarrhea, and promotion of the bacterial dysbiosis associated with this diarrhea. After all, there is already the precedent that the canine respiratory coronavirus (CRCoV) affects the dog gastrointestinal tract [28].

The virus found in the feces of this dog by RT-PCR amplification, partial Sanger sequencing of the *S* gene and almost complete NGS genome sequencing, is clearly proven to be SARS-CoV-2 by clustering of this sequence, in a molecular phylogenetic tree, with SARS-CoV-2 virus variants, whereas it was more distant from other human or canine coronaviruses. Furthermore, it was classified as B.1.177 variant. This variant has circulated widely in many countries of Europe including Spain [25], being the most prevalent SARS-CoV-2 variant in the Balearic Islands between August 2020 and February 2021 [29], the period in which the dog of this study was infected (January 2021).

Thus, from the antecedent of human infection in the family hosting this pet dog and the intense contact between Ceuta and peninsular Spain, the dog likely was infected from his human hosts, who may have been infected by the then widely circulating in Spain B.1.177 variant. Unhappily, the SARS-CoV-2 virus that infected the humans cohabiting with this pet was not sequenced, in line with the low viral genomic sequencing in Ceuta in 2020 and early 2021 [30, 31] as well as in Morocco in the same period [32].

On the other hand, the Ile402Val substitution was apparently unsuccessful in spreading, given its lack of widespread reporting until now, more than one and a half years after its occurrence in the fecal specimen analyzed here. This is attested by the fact that, among the almost 11 million sequences collected in the GISAID database, this substitution has only been detected in 317 sequences (including the one from our dog). It is true that, in principle, the Ile402Val substitution, because it maps in the RBD, could affect virus/receptor interactions. However, the mildness of the amino acid substitution involved may dispel the possibility of a drastic effect. Both Ile and Val are branched hydrophobic aliphatic amino acids differing only in one carbon length in their side chains, leading to the consideration of the Ile>Val substitutions as a conservative replacement. This would agree with the lack of success in spreading of viral variants hosting this substitution, finally making unlikely the conceivable possibility that this substitution is a change of concern.

In conclusion, our results not only add another example for the view that dogs can be SARS-CoV-2-infected when intimately related with COVID-19 affected humans, but they also provide evidence of B.1.177 variant circulation in a North African city in a period for which data were very limited in the city’s region. We also virtually exclude that the Ile402Val substitution in the S protein present in the virus infecting this dog, which conceivably could be a variant of concern, actually posed a particular threat. The presentation of clinically relevant hemorrhagic diarrhea calls for further studies to evaluate the implication of SARS-CoV-2 in this canine pathology.

## Supplementary Information

**Additional file 1.** Supplementary methods.

## Data Availability

The partial 399-bp sequence of SARS-CoV-2 *S* gene detected in the dog’s stool sample has been deposited in the NCBI GenBank under the Accession Number MW872017 and in the GISAID database with the Accession ID EPI_ISL_1490671. The complete genome sequence was also submitted to GISAID (Accession ID EPI_ISL_12695538). Finally, all raw sequencing data from the dog’s stool sample obtained by NGS have been submitted to the NCBI Sequence Read Archive (SRA) repository, under the BioProject accession number PRJNA810957.

## Abbreviations

ACE2: Angiotensin-converting enzyme 2
AHD: Acute hemorrhagic diarrhea
CBC: Complete blood cell count
COVID-19: Coronavirus disease 2019
CRT: Capillary refill time
HCoV-HKU1: Human coronavirus HKU1
HCoV-OC43: Human coronavirus OC43
IBD: Inflammatory bowel disease
INCLIVA: Health Research Institute of the Hospital Clínico de Valencia
MERS-CoV: Middle East respiratory syndrome coronavirus
NGS: Next-generation sequencing
NHD: Acute non-hemorrhagic diarrhea
RBD: Receptor binding domain
RT-PCR: Reverse transcription polymerase chain reaction
SARS-CoV: Severe acute respiratory syndrome coronavirus
SARS-CoV-2: Severe acute respiratory syndrome coronavirus 2
SARSr-CoV: Severe acute respiratory syndrome related coronavirus
