# Supplementary material for: Detection of SARS-CoV-2 in a dog with hemorrhagic diarrhea
Source: BMC Vet Res. 2022 Oct 12;18:370. doi: 10.1186/s12917-022-03453-8 (PMC9554378; doi:10.1186/s12917-022-03453-8)
Supplement: Supplementary file 1 — Additional file 1. Supplementary methods. [file 12917_2022_3453_MOESM1_ESM.docx]

**Detection of SARS-CoV-2 in a dog with hemorrhagic diarrhea**

Miguel Padilla-Blanco^a^, Santiago Vega^b*^, Luis Enjuanes^c^, Alfonso Morey^d^, Teresa Lorenzo^e^, Clara Marin^b^, Carmen Ivorra^f^, Elisa Maiques^e^, Vicente Rubio^g*^, Consuelo Rubio-Guerri^a*^

***Corresponding authors:** [svega@uchceu.es](mailto:svega@uchceu.es) (S. Vega), rubio@ibv.csic.es (V. Rubio), [consuelo.rubio@uchceu.es](mailto:consuelo.rubio@uchceu.es) (C. Rubio-Guerri)

**Affiliations**

^a^ Departamento de Farmacia, Facultad de Ciencias de la Salud, Universidad Cardenal Herrera-CEU, Alfara del Patriarca (Valencia), Spain.

^b^ Departamento de Producción y Sanidad Animal, Salud Pública Veterinaria y Ciencia y Tecnología de los Alimentos, Instituto de Ciencias Biomédicas, Facultad de Veterinaria, Universidad Cardenal Herrera-CEU, Alfara del Patriarca (Valencia), Spain.

^c^ Centro Nacional de Biotecnología (CNB-CSIC), Campus de Cantoblanco, Universidad Autónoma de Madrid, 28049 Madrid, Spain.

^d^ Clínica Vetererinaria Morey, Ceuta, Spain.

^e^ Departamento de Ciencias Biomédicas, Facultad de Ciencias de la Salud, Universidad Cardenal Herrera-CEU, Alfara del Patriarca (Valencia), Spain.

^f^ I+D+I Department, Sequencing Multiplex SL (I+D+I, Seqplexing), Parque Científico Universidad de Valencia, Paterna (Valencia), Spain.

^g^ Instituto de Biomedicina de Valencia del Consejo Superior de Investigaciones Científicas (IBV-CSIC) and CIBERER-ISCIII, Valencia, Spain.

**Materials and methods**

**Detection of SARS-CoV-2 in dog faeces**

A small amount of faeces aseptically collected by a COVID-19-free veterinary technician were placed in a 1.5 ml sealed plastic tube with 200 µL of RNA preservation/viral inactivating fluid [1] and were frozen and sent in dry ice to the analysing laboratory in Valencia, for testing the presence of SARS-CoV-2. They were kept at -80ºC until use (<10 days). After thawing in ice, RNA was isolated in 50 μL of RNase-free water using a commercial kit (Total RNA Isolation kit, NZYtech, Portugal) and RNA was subjected to a two-step RT-PCR to amplify the SARS-CoV-2 viral spike glycoprotein gene (*S*) as recently reported [2]. In short, cDNA was generated and used for qPCR-amplification of a 399-nt region (nucleotides 22727-23125) of the *S* gene. Positivity was proven by fluorescence output of qPCR, by agarose gel electrophoresis of the amplified products and by Sanger DNA sequencing. Contamination was excluded, and the positive control used was the cDNA of the B.1.258 variant of SARS-CoV-2, as reported in [2]. For Sanger sequencing of the qPCR-amplified *S* fragment and for one-tube commercial RTqPCR assay (Viasure, fromCerTestBiotec, Zaragoza, Spain), see [2]. The *S* gene was aligned with retrieved sequence data to determine the mutations present in this region (nucleotide and inferred amino acid sequences), using BioEdit, v.7.2.5 [3].

**NGS sequencing and bioinformatic analysis with SARS-CoV-2 reads**

NGS sequencing was done by the Sequencing Multiplex SL Valencia spinoff of the Health Research Institute of the Hospital Clínico de Valencia (INCLIVA). RNA was retrotranscribed to cDNA using random hexameric oligonucleotides and SuperScript IV reverse transcriptase (ThermoFisher) following the manufacturer’s instructions. DNA library was prepared utilising Artic v3 enrichment by Nextera DNA Flex Library prep (Illumina). Sequencing-ready libraries were then loaded onto the Illumina MiSeq platform and a 150-bp paired-end sequencing kit was used (Illumina, Inc., San Diego, CA, USA). Separate FASTQ files used for forward and reverse reads contained 311,898 reads each. The Galaxy platform (<https://usegalaxy.org/>) [4] was used to perform the bioinformatic analyses. Briefly, quality of raw reads was visualized by using the FASTQC software. Next, Cutadapt software was used to filter the raw reads according to their length and their quality, remaining only those having ≥ 100 bp length and at least Q20 quality (99% base-call accuracy). High quality of the filtered reads (hereinafter referred as high quality reads) was proved using again the FASTQC tool. After the filtering procedure, high quality reads were aligned against the SARS-CoV-2 reference genome (GenBank Accession Number NC_045512.2), using the BWA-MEM aligner. Only reads which were in both the forward and reverse FASTQ files were utilized for the alignment, given that we used a paired-end strategy. By the combination of Samtools and Bcftools, we obtained the sequencing depth for each position (number of times each position had been mapped) and detected the mutations present. Those positions which presented 0 sequence depth were manually converted to undetermined nucleotides (Ns) The whole obtained sequence was submitted to GISAID, which determines the SARS-CoV-2 variant to which the genome belongs.

**Phylogenetic analysis**

Molecular phylogenetic analysis was carried out as reported [2], using for analysis the near-complete genome sequence obtained by NGS, utilizing MEGA11 software [5]. To facilitate automated analysis, all the Ns of the present SARS-CoV-2 sequence and of the other sequences utilized in this analysis were substituted by the corresponding nucleotides of the consensus genome sequence. P-distance matrices were calculated, and tree topology was deduced by the Neighbour-Joining method based on p-distance (bootstrap on 2000 replicates, generated with a random seed).

**Bioinformatic analysis with non-SARS-CoV-2 reads**

The high-quality reads which did not map against the SARS-CoV-2 genome were also aligned to elucidate the organism to which they belonged. Using STAR aligner [6] the dog genome-aligning reads were identified and removed from the FASTQ file. The remaining reads were classified according to their taxonomy (unclassified, domain, kingdom, phylum, class, order, family, genus or species) with the help of Kraken 2 (<https://ccb.jhu.edu/software/kraken2/>, version 2.0.8-beta; maxikraken2_1903_140GB, database, which contains archaeal, bacterial, fungal, protozoal, viral and human genomes) [7,8] and of Recentrifuge (<https://www.recentrifuge.org>, release 1.3.3) [9]. The estimation of the abundance of organisms of each phyla corresponds to the relative abundance of reads assigned to microorganisms of each of these phyla.

**Supplementary references**

**References**

1. Padilla-Blanco M, Aguiló-Gisbert J, Rubio V, Lizana V, Chillida-Martínez E, Cardells J, et al. (2022) The finding of the severe acute respiratory syndrome coronavirus (SARS-CoV-2) in a wild Eurasian river otter (*Lutra lutra*) highlights the need for viral surveillance in wild mustelids. Front Vet Sci 9:826991.
2. Aguiló-Gisbert J, Padilla-Blanco M, Lizana V, Maiques E, Muñoz-Baquero M, Chillida-Martínez E, et al. (2021) First description of SARS-CoV-2 infection in two feral American mink (*Neovison vison*) caught in the wild. Animals (Basel) 11:1422.
3. Hall TA (1999) BioEdit: a user-friendly biological sequence alignment editor and analysis program for Windows 95/98/NT. Nucleid Acids Symp Ser 41:95-98.
4. Afgan E, Baker D, Batut B, van den Beed M, Bouvier D, Cech M, et al. (2018) The Galaxy platform for accessible, reproducible and collaborative biomedical analyses: 2018 update. Nucleic Acids Res. 2018;46:W537-44.
5. Tamura K, Stecher G, Kumar S (2021) MEGA11: molecular evolutionary genetics analysis version 11. Mol Biol Evol 38:3022-7.
6. Dobin A, Davis CA, Schelesinger F, Drenkow J, Zleski C, Jha S, et al. (2013) STAR: ultrafast universal RNA-seq aligner. Bioinformatics 29:15-21.
7. Wood DE, Salzberg SL (2014) Kraken: ultrafast metagenomic sequence classification using exact alignments. Genome Biol 15:R46.
8. Wood DE, Lu J, Langmead B (2019) Improved metagenomic analysis with Kraken 2. Genome Biol 20:257.
9. Martí JM (2019) Recentrifuge: robust comparative analyses and contamination removal for metagenomics. PLoS Comput Biol 15:e1006967.
